# 891. Cytomegalovirus Testing Rates Among Immunocompromised Adults, Adolescents, And Children In The United States

**DOI:** 10.1093/ofid/ofad500.936

**Published:** 2023-11-27

**Authors:** Purva Jain, Sandeep Basnet, Sahar Syed, Brent Arakaki, Katherine E Mues, Zachary Marcum, John D Diaz-Decaro

**Affiliations:** Aetion, Inc., New York City, New York; Moderna, Inc., Cambridge, Massachusetts; Aetion, Inc., New York City, New York; Aetion, Inc., New York City, New York; Aetion, Inc., New York City, New York; Aetion, Inc., New York City, New York; Moderna, Inc., Cambridge, Massachusetts

## Abstract

**Background:**

Cytomegalovirus (CMV) is a latent virus that can cause morbidity and mortality among immunocompromised (IC) individuals. No studies have examined how CMV testing changed throughout the COVID-19 pandemic among IC individuals in the United States (US). This study aimed to estimate CMV testing rates among IC adults (18-94 years), adolescents (12-17 years), and children (0-11 years) during the COVID-19 pandemic.

**Methods:**

This observational cohort study included insured individuals from the US and was conducted using administrative medical and pharmacy claims from HealthVerity from December 2017-May 2022. IC adults, adolescents, and children entered the cohort after 6 months of continuous enrollment and were followed until disenrollment or end of study period. CMV testing was captured using CPT codes (serology and PCR) and was limited to no more than 1 test per day with ≥6 days required between tests. The rate of CMV testing (per 100,000 person-months) was estimated over three time periods defined by key points in the COVID-19 pandemic: pre-COVID-19-era, 06/01/2018-03/31/2020; pre-vaccine-COVID-19-era, 04/01/2020-12/31/2020; and vaccine-COVID-19-era, 01/01/2021-05/31/2022.

**Results:**

The sample included 735,251 IC adults, of whom 4.8% (N=35,391) had ≥1 CMV test over follow-up (median follow-up, 566 days); 12,203 IC adolescents, of whom 15.5% (N=1,896) had ≥1 CMV test over follow-up (median follow-up, 582 days); and 15,854 IC children, of whom 14.7% (N=2,324) had ≥1 CMV test over follow-up (median follow-up, 702 days). The mean rate of CMV tests per 100,000 person-months for IC adults was 1,660 in the pre-COVID-19-era, 1,040 in the pre-vaccine-COVID-19-era, and 983 in the vaccine-COVID-19-era. Similarly, rates of CMV testing declined among IC adolescents and children (adolescents: 7,391 in pre-COVID-19-era, 4,717 in pre-vaccine-COVID-19-era, and 4,219 in vaccine-COVID-19-era; children: 7,556 in pre-COVID-19-era, 4,467 in pre-vaccine-COVID-19-era, and 4,026 in vaccine-COVID-19-era). The highest testing rates were among those with a history of stem cell transplantation in all age cohorts.

**Conclusion:**

CMV test utilization decreased during the COVID-19 pandemic era in the US and remained low through the early COVID-19 vaccine era.

**Disclosures:**

**Sandeep Basnet, MD**, Moderna Therapeutics: Stocks/Bonds **Brent Arakaki, BS**, Aetion, Inc.: Employee **Katherine E. Mues, PhD**, Aetion, Inc.: Employee **John D. Diaz-Decaro, PhD, MS**, Moderna Therapeutics: Stocks/Bonds

